# Design of a Microsphere-Based High-Throughput Gene Expression Assay to Determine Estrogenic Potential

**DOI:** 10.1289/ehp.7843

**Published:** 2005-05-12

**Authors:** Jorge M. Naciff, Brian D. Richardson, Kerry G. Oliver, M. Lynn Jump, Suzanne M. Torontali, Kenton D. Juhlin, Gregory J. Carr, Jennifer R. Paine, Jay P. Tiesman, George P. Daston

**Affiliations:** 1 Miami Valley Innovation Center, Procter & Gamble Company, Cincinnati, Ohio, USA; 2 Radix BioSolutions, Georgetown, Texas, USA

**Keywords:** endocrine disruptor, estrogen-regulated gene expression, fluorescently coded microspheres, high-throughput gene expression assay, multiplex

## Abstract

Recently gene expression studies have been multiplied at an accelerated rate by the use of high-density microarrays. By assaying thousands of transcripts at a time, microarrays have led to the discovery of dozens of genes involved in particular biochemical processes, for example, the response of a tissue/organ to a given chemical with therapeutic or toxic properties. The next step in these studies is to focus on the response of a subset of relevant genes to verify or refine potential therapeutic or toxic properties. We have developed a sensitive, high-throughput gene expression assay for this purpose. In this assay, based on the Luminex xMAP system, carefully selected oligonucleotides were covalently linked to fluorescently coded microspheres that are hybridized to biotinylated cRNA followed by amplification of the signal, which results in a rapid, sensitive, multiplexed assay platform. Using this system, we have developed an RNA expression profiling assay specific for 17 estrogen-responsive transcripts and three controls. This assay can evaluate up to 100 distinct analytes simultaneously in a single sample, in a 96-well plate format. This system has improved sensitivity versus existing microsphere-based assays and has sensitivity and precision comparable with or better than microarray technology. We have achieved detection levels down to 1 amol, detecting rare messages in complex cRNA samples, using as little as 2.5 μg starting cRNA. This assay offers increased throughput with decreased costs compared with existing microarray technologies, with the trade-off being in the total number of transcripts that can be analyzed.

The effectiveness of characterizing changes in gene expression in response to chemical exposure, therapeutic or toxic, or caused by changes in physiological status of tissues or organs (disease, development, etc.), has grown exponentially in the past few years because of the availability of cost-effective microarray technology. Microarrays allow the quantitative analysis of thousands of gene expression changes simultaneously in a single experiment, orders of magnitude more than the number that could be evaluated using older technologies for quantitative gene expression [i.e., Northern blot, RNase protection, quantitative real-time polymerase chain reaction (QRT–PCR)]. This approach has led to the discovery of multiple genes putatively involved in particular biochemical processes. Once a subset of relevant genes has been identified, the next step usually focuses on the response of that subset, for example, to confirm or refine potential therapeutic or toxic targets. This step has been performed largely using traditional methods, namely, QRT–PCR or Northern blot analysis. However, these methods are time- and sample-consuming because of their one-gene-at-a-time approach. What is needed is an intermediate technology to evaluate changes in the expression level of 10–100 genes (multiplexed), with high specificity, sensitivity, and reproducibility.

In previous studies using high-density oligonucleotide arrays, we have shown that exposure to various estrogen receptor (ER) agonists induced a characteristic gene expression profile in the developing reproductive system of the female rat (Naciff et al. 2002, 2003). Among these genes, there is a subset whose changes in expression are specific for estrogen exposure. Using this subset of genes (molecular fingerprint) rather than a single biomarker, we have developed a high-throughput gene expression assay based on the Luminex xMAP system (Luminex Corp., Austin, TX). The Luminex system is a multi-analyte bioassay detection system capable of performing up to 100 assays simultaneously in a single microtiter plate well. This system uses polystyrene microspheres internally dyed with red and infrared fluorophores that can be individually identified. The fluorescent microspheres can be coated with a reagent specific to a particular bioassay, allowing the capture and detection of specific analytes from a sample. This assay has been used to quantify proteins, genotype patients, and test viral loads in a multiplex platform (Brodsky and Silver 2002; Dunbar et al. 2003; Fulton et al. 1997; Gordon and McDade 1997; Morgan et al. 2004; Oliver et al. 1998; Smith et al. 1998; Vignali 2000). To date, there are many kits available to measure different proteins (Earley et al. 2002; Luminex 2005) in different samples based on the Luminex microspheres. However, there is only a single reference in the literature describing the use of this approach to quantify gene expression at the level of RNA transcripts (Yang et al. 2001). These authors have shown the ability to detect the expression level of up to 20 genes simultaneously with a lower detection limit of 100 amol. This detection limit allows the evaluation of only the moderately to highly expressed genes, a limitation to its broader applicability. We report here significant improvements to the assay that increases the sensitivity by two orders of magnitude, down to a single attomole of labeled target in a complex target mixture (for comparison, the detection limit of the Affymetrix microarray chip is about 15 amol per transcript). The assay we have developed is suitable for detection of up to 100 different transcripts with high throughput of hundreds to thousands of samples per day, with high accuracy, speed, sensitivity, and flexibility (add or subtract specific transcripts). It is particularly valuable for applications requiring the detection of a moderate number of transcripts in thousands of samples.

Our necessity for a high-speed multiplex assay has been driven by an imminent U.S. Environmental Protection Agency (EPA) requirement to determine the potential for the chemicals it regulates to interact with estrogen, androgen, or thyroid hormone system (U.S. EPA 2005). We believe that gene expression analysis has the greatest potential to evaluate these interactions in a sensitive and specific manner and is most amenable to the development of *in vitro* alternatives. We have used microarray analysis to determine changes in gene expression (to identify a “molecular fingerprint”) induced by estrogens, including the potent ER agonist, 17α-ethynyl estradiol (EE), in the female reproductive system of the rat. Previous studies in this laboratory have shown that transplacental exposure to various ER agonists (genistein, bisphenol A, and EE), or direct exposure (in prepubertal female rats) elicited a specific profile of gene expression changes in response to these chemicals (Naciff et al. 2002, 2003). From the set of affected genes, we have chosen a subset for which expression is strongly influenced by estrogen exposure that was used as a test case for evaluating the performance of the microsphere-based assay.

## Materials and Methods

### Animals, treatments, and target preparation.

Total RNA was isolated and purified according to the method previously reported by this laboratory (Naciff et al. 2003). Briefly, 15-day-old Sprague-Dawley female rats (Charles River, Raleigh, NC) were obtained and housed for 5 days before treatment. The experimental protocol was carried out according to Procter & Gamble’s approved protocols for animal care, and animals were maintained in accordance with the National Institutes of Health *Guide for the Care and Use of Laboratory Animals* (Institute of Laboratory Animal Resources 1996). On day 20 the rats were treated with either vehicle (peanut oil) or 0.1, 1.0, or 10.0 μg/kg EE each day for 4 consecutive days. This dose regimen was selected to elicit a graded (dose-dependent) uterotrophic response in the immature rat as indicated in Naciff et al. (2003). On day 24 the rats were sacrificed. The reproductive tissues (uteri with ovaries attached) were excised, trimmed of fat and connective tissue, and stored in RNAlater (Ambion, Inc., Austin, TX) at 4°C. We chose to evaluate the uterus and ovaries as a pool because they are two of the tissues most sensitive to estrogenic regulation. Although we realize that this may result in loss of information about gene expression in one or the other organ, we believe that from a screening perspective we have taken the best approach because these organs contain considerable variation in the expression levels of the two ER isoforms, ER-α and ER-β, and consequently have the potential to represent gene expression changes induced by activation of any of the isoforms of the ER in the target tissues. RNA was isolated using TriReagent (Molecular Research Center, Inc., Cincinnati, OH). Total RNA was further purified using the RNeasy Kit (Qiagen Inc., Valencia, CA) and stored at −80°C. Ten micrograms of RNA, from five independent samples (biologic replicates), was then converted into double-strand cDNA. The Enzo Bioarray RNA Transcription Kit (Affymetrix Inc., Santa Clara, CA) was then used to generate 50–75 μg of biotin-labeled cRNA. The cRNA was fragmented and hybridized to Affymetrix rat 34A chips. Unused fragmented cRNA was stored at −20°C until use for these experiments. The batch of cRNA was used in the many steps of optimizing the assay until none was left. The data shown here in the final assay are from a new batch of cRNA generated from the original stock of total RNA, which was used to generate the cRNA evaluated by microarray analysis. To fully assess the overall quality of each sample, the newly labeled cRNA samples were hybridized to the Affymetrix GeneChip Test 3 Array as previously described (Naciff et al. 2003).

### Oligonucleotides.

To verify the data from the microarray experiments, we chose to use a single oligonucleotide to confirm the data from 16–20 oligos that are tiled for each feature on the U34A rat chips (for a full description of the rat U34A array content, see Affymetrix 2005). Briefly, in the rat genome U34A high-density oligonucleotide microarray, each gene or expressed sequence tag (EST) is represented by 16–20 pairs of 25-mer oligonucleotides that span the coding region. Each probe pair consists of a perfect match sequence that is complementary to the cRNA target and a sequence that is mismatched by a single base change at the middle of the nucleotide, a region critical for target hybridization. The mismatched oligonucleotide serves as a control for non-specific hybridization. We hypothesized that one probe could be used for the evaluation of the corresponding transcript. To identify the best possible oligonucleotide (probe), we designed an algorithm that allowed us to select the best-performing probe (oligonucleotide) from the entire probe set. This algorithm evaluates different parameters that best predict the results that were obtained from all probes in the feature. Another option was to select the best two or even three probes. Thus, the second- and third-best-performing probes, for each transcript selected, were identified using the different parameters evaluated by the algorithm. To test the validity of our approach, two oligonucleotides were identified as being the best-performing probes to evaluate the mRNA level for the rat glyceraldehyde 3-phosphate dehydrogenase (*GAPDH*). Additionally, because Affymetrix chips employ a set of perfect match–mismatch pair of oligonucleotides tiled onto the microarray to better evaluate the specific signal of any given mRNA, relative abundance, we decided to apply this strategy to the microsphere assay. To test this approach, a perfect match and a mismatch probe for 11β-hydroxysteroid dehydrogenase (*Hsd11b2*) were identified and used for the evaluation of the mRNA level of *Hsd11b2* in the different samples. Empirical determination of the value of using the mismatch probe, paired with the data obtained from the perfect match probe, indicated that the specificity of the signal was not improved by its use (data not shown). The signal values from the perfect match probe are highly specific and are the only ones reported. The different probes selected are indicated in Table 1.

### Coupling of oligonucleotides to microspheres.

Transcript-specific oligonucleotides, corresponding to the complementary sequence of the desired mRNA (Table 1), were covalently coupled to fluorescently distinct sets of carboxylate-modified polystyrene xMAP microspheres using water-soluble carbodiimide. All oligonucleotides were purchased from Integrated DNA Technologies Inc. (Coralville, IA) and were synthesized with a 5′-amino linker and a C12 spacer. These oligos were coupled covalently to 20 fluorescently distinct sets of carboxylated microspheres as previously detailed (Fulton et al. 1997) at a ratio of 1 nmol of modified oligo to 1 × 10^7^ microspheres. The 20 sets of carboxylated polystyrene microspheres (5.6 μm in diameter) of differing fluorescence addresses (determined by its spectral signature determined by its red/infrared fluorophore ratio) were ordered from Luminex Corp. Specifically, 1 × 10^7^ microspheres were pelleted in a microcentrifuge (12,000 × *g*) for 2 min, and the supernatant was carefully removed. The microspheres were resuspended in 50 μL of buffer containing 0.1 M MES [2-(*N*-morpholino)ethanesulfonic acid; Sigma-Aldrich Co., St. Louis, MO] at pH 4.5. The amino-substituted oligonucleotides were dissolved in double-distilled H_2_O to 1 mM and stored at −80°C. For the coupling reaction, 1 nmol of the appropriate oligonucleotide was added to the desired microsphere set. The reaction was initiated by adding 2.5 μL of freshly prepared 10 mg/mL 1-ethyl-3-(3-dimethyl-aminopropyl) carbodiimide hydrochloride (EDC; Pierce, Rockford, IL), followed by incubation of the mixture for 30 min at room temperature, in the dark. After the initial 30 min incubation, a second 2.5 μL of freshly prepared EDC solution (10 mg/mL) was added to the reaction and incubated for an additional 30 min. The coupling reaction was terminated by adding 1 mL of 0.02% Tween 20 (polyoxyethylenesorbitan monolaurate; Sigma-Aldrich Co.) to the microsphere suspension, vortexed, and then centrifuged (12,000 × *g*) for 4 min; the supernatant containing free oligonucleotides and unreacted EDC was removed. To ensure that all the uncoupled oligonucleotides were removed, the microspheres were washed with 1 mL of 0.1% SDS (Sigma-Aldrich Co.). The oligonucleotide-conjugated microspheres were resuspended in 1 mL of TE buffer (10 mM Tris, 1 mM EDTA, pH 8.0; Sigma-Aldrich Co.), counted on a hemacytometer, adjusted to a concentration of 1 × 10^7^ microspheres/mL, and stored in TE at 4°C, protected from the light. Complementary sense-strand oligos were ordered with a 5′ biotin modification to be used for titration curves for each set of the coupled microspheres.

### Hybridization.

To optimize the system, initial experiments used biotinylated complementary sense-strand probes to develop a titration curve to examine the sensitivity of the assay. In this assay five genes (analytes) were chosen to evaluate the sensitivity of this system. To this end 5,000 microspheres per analyte were combined in a well of a 96-well plate containing 1× TMAC [3 M tetramethyl-ammonium chloride, 0.1% SDS; 50 mM Tris–HCl, pH 8.0; and 4 mM EDTA, pH 8.0; Sigma-Aldrich Co.] in a total volume of 50 μL. Biotinylated complementary oligonucleotide (4 μL) at varying concentrations (one-half log serial dilutions for 100 fmol to 100 amol) diluted in TE (10 mM Tris and 1 mM EDTA, pH 8.0) was added and mixed. Samples were heated at 95°C for 2 min. The mixture was transferred to a 48°C shaking heat block (Eppendorf Thermomixer; Eppendorf North America, Inc., New York, NY) for 1 hr. The samples were spun at 2,500 × *g*, and the supernatant was removed by inverting the plate over the waste container and tapping one time on a table covered with absorbent paper. Samples were washed once with 1× TMAC and resuspended with 65 μL streptavidin–phycoerythrin (SAPE; Molecular Probes, Eugene, OR) stain (5 μg/mL SAPE in 1× TMAC) for 15 min at room temperature. The samples were then analyzed on a Luminex 100 instrument (Luminex Corp.).

For the amplification of the signal (see “Results”), the following steps were added to the protocol. After the washes after the SAPE stain, the microspheres were incubated for 60 min with biotinylated anti-streptavidin antibody (Vector Laboratories, Burlingame, CA) with goat IgG (Sigma-Aldrich Co.) to block nonspecific binding. The microspheres were spun, the supernatant was removed, and the microspheres were then stained again (Table 4) with 50 μL SAPE for 10 min. The microspheres were then washed a final time with PBS–1% BSA and analyzed.

Through empiric determination, the optimized protocol (Table 4) has incorporated the following changes: 1× TMAC was replaced with 0.5× TMAC, the two SAPE incubations and the anti-streptavidin incubation steps were performed with PBS–1% BSA as the buffer; and the bead concentration was reduced 10-fold. Because the flow cytometric analysis is done at a preset photomultiplier (PMT) setting, there is the possibility that when a given transcript is relatively abundant (high copy number), the signal values could reach saturation, whereas the signal values derived from the hybridization of uncommon transcripts (low copy number) to their specific microspheres could be relatively low. To avoid this potential problem, and taking advantage of the instrument used in our studies [Luminex 100 system from Luminex Corp. or Bio-Plex Suspension Array System from Bio-Rad Laboratories, Inc. (Hercules, CA)], we analyzed the fluorescence signal values at two PMT settings (low and high) to expand the dynamic range of the assay without losing sensitivity.

### Instrumentation.

Samples run at Radix BioSolutions were read on a Luminex 100 (SP1 software, version 1.7.69; Luminex Corp.). Samples run at Procter & Gamble’s Miami Valley laboratories were read on the Bio-Rad Bio-Plex (Bio-Rad’s version of the identical Luminex 100 flow cytometer) using the Bio-Plex Manager software, version 2.0. Each system integrates lasers, optics, fluidics, electronics, and signal processing to identify each set of fluorescent-coded microspheres from each set and to measure the total fluorescence at the surface of each microsphere to quantify the amount of reporter bound to it. The median fluorescence intensity (MFI), derived from reading at least 100 microspheres from each set, was used as a representation of the whole population of microspheres of each set in any given sample. The MFI from five independent samples (biological replicas), from control or from each EE-treated animal, were determined and used to determine the average fold change on the expression of the transcript of interest.

### QRT–PCR.

To verify the relative change in gene expression identified by both the microspheres and the oligonucleotide microarrays, we used a real-time (kinetic) QRT–PCR approach as previously described by this laboratory (Naciff et al. 2002, 2003). The primers used for the QRT-PCR have also been published (Naciff et al. 2003).

## Results

### Design of the transcript quantification assay.

Our goal was to quantify the amount of specific transcripts present in a complex mixture of cRNA with a sensitivity and specificity comparable with at least those obtained from the microarray analysis. To this end we chose oligonucleotides with unique sequences of 25 bases in length, based upon the best-performing probes tiled on the Affymetrix rat genome U34A high-density oligonucleotide microarray. Effective oligonucleotide probes for each transcript were selected based on the data from 44 chip hybridizations (Naciff et al. 2003), using a statistical algorithm (Juhlin KD, Carr GJ, Jump ML, Richardson BD, Torontali SM, unpublished data), and the analyzed data. The expression of 17 of the 20 target transcripts evaluated in this article is regulated by estrogens in a dose-dependent manner. We also included two internal control transcripts (vascular α-actin and cyclophilin B) that are also expressed in the uterus/ovaries of the immature rat but whose expression is not regulated by estrogens in these organs. An oligonucleotide specific for a bacteriophage M13 gene was also included as an external control. The sequences of the oligonucleotides are shown in Table 1.

### Sensitivity and specificity of hybridization of the selected oligonucleotides coupled to fluorescent microspheres.

Each of the 20 oligonucleotides, derived from the 19 *Rattus norvegicus* genes and a control bacteriophage M13, was coupled to xMAP microspheres with a unique fluorescence emission wavelength. Each microsphere set was evaluated individually for specificity and sensitivity, after which the different microsphere sets were combined for multiplexed assays.

**Figure 1:**
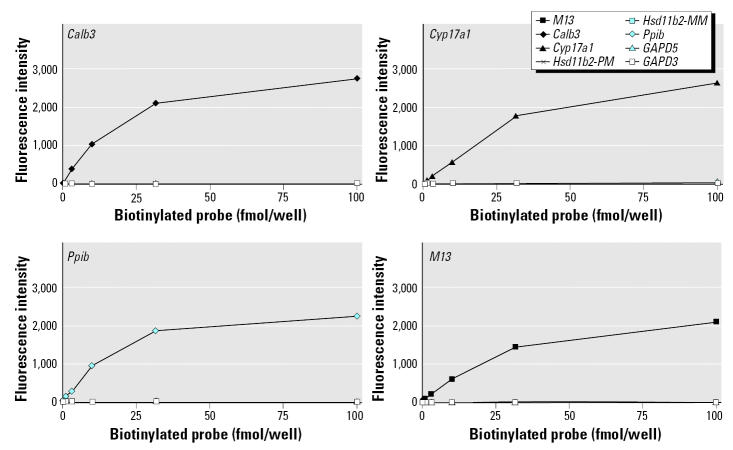
Sensitivity and specificity of the assay. To determine the specificity and sensitivity of the assays, a series of multiplexed hybridization reactions were performed. Four different biotinylated complementary oligonucleotides (*Calb3, M13, Cyp17a1*, and *Ppib*) were hybridized at different concentrations (0, 0.1, 0.316, 1, 3.16, 10, 31.6, 100 fmol) to eight different sequence-specific microsphere sets. The data demonstrate undetectable cross-hybridization. *Calb3,* intestinal calcium-binding protein, or calbindin 3; *Cyp17a1,* 17α-hydroxylase cytochrome P450; *Ppib,* cyclophylin B or peptidylprolyl isomerase B.

To determine the specificity and sensitivity of the assays and to optimize the system, initial hybridization experiments used a titration of biotinylated complementary sense-strand probes. Five specific microspheres sets were used to evaluate the sensitivity of this system. A complex mixture of different concentrations of synthetic complements, which included the antisense oligonucleotides specific for *M13*, *Calb3*, *Cyp171a*, *Pp1b*, and *Hsd11b2* (sequences are given in Table 1), was mixed with oligonucleotides specific to *GAPDH*-5′ and *GAPDH*-3′ mRNA region (5′-CGTCAAGATCAAATGGGGTGAT GCT-3 and 5-ATCCTGGGCTACACT GAGGACCAGG-3′, respectively), and a single-base mismatch for *Hsd11b2* (mutation on the central region, 5′-TCATGAGACC ATcTATACCCTACC-3′). Oligonucleotide-coupled microspheres were combined with different concentrations of biotinylated complementary oligonucleotides and allowed to hybridize at 48°C. The signal of each analyte bound to each type of microsphere present in the reaction was determined using SAPE as a reporter fluorescent tag. A negative control consisted of all assay reactants except for the biotinylated complementary oligonucleotides. The median fluorescent intensity of at least 100 microspheres of each set was calculated by the software. The intensity of the hybridization signals is linearly related to the amount of analyte being evaluated, from 0 to 36.1 fmol, for each oligonucleotide–microsphere set (Figure 1). The assay was no longer linear at higher concentrations of analyte (100 fmol). The specificity of the hybridization is not compromised as the number of analytes increases.

### Evaluation of the sensitivity and specificity of the assay in experimental samples.

The transcripts to be analyzed (analytes or targets) were biotin-labeled cRNAs, prepared from the uterus/ovaries of prepubertal rats exposed to vehicle control or various doses of EE for 4 days (0.1, 1, or 10 μg EE/kg/day, low, mid, or high doses, respectively, *n* = 5 for each dose group), collected from a previous study (Naciff et al. 2003). Each sample from every dose group was analyzed independently and used as a biological replicate.

**Figure 2:**
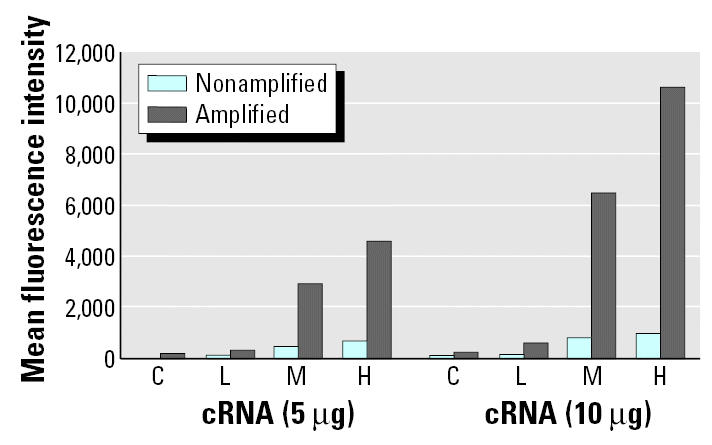
Comparison of the quantification of *Calb3* expression with and without signal amplification. Biotinylated cRNA samples from control (C) vehicle-treated or EE-exposed tissues (0.1, 1.0, or 10.0 μg/kg/day; L, M, and H, respectively) were hybridized to microspheres coupled to the specific probe for *Calb3,* and the fluorescent intensity of at least 100 microspheres was determined (nonamplified). To increase the signal value, two equivalent sets of samples were hybridized to the specific *Calb3* microspheres, washed, and then hybridization signal was amplified as indicated in ”Materials and Methods.” A clear dose response to EE exposure can be determined in both cases; however, the amplification step increases the sensitivity of the assay.

Initial sensitivity results of the microsphere-based assay were not within the range needed for the detection of all transcripts. The hybridization signal from one transcript, intestinal calcium-binding protein (*Calb3*), is shown in Figure 2. Although the assay clearly allows for the determination of the changes induced in *Calb3* by EE exposure (control vs. low, mid, or high doses; C, L, M, and H respectively), the corresponding signal values were near the lower end of the sensitivity. The signal intensities obtained were similar to those obtained by Yang et al. (2001). The fluorescence intensity (signal value) corresponding to the transcript levels of *Calb3* in the control sample (vehicle treated) was extremely low (44 units with 5 μg cRNA and 68 units with 10 μg cRNA). *Calb3* is known to be a gene transcript that shows a moderately high expression in control samples and is stimulated to high expression levels with high doses of EE as shown by Affymetrix GeneChip data (4,700 units in control to 49,400 units in high-dose group with a chip scaling factor of 1,500). Although the fluorescence signal had high specificity (Figure 1) and it was well above the background level (the fluorescence of the microspheres not exposed to cRNA), in the context of a very complex cRNA background the small signal intensity left little room for the detection of gene transcripts that were expressed less abundantly than *Calb3*.

### Signal amplification using biotin-labeled anti-streptavidin antibody.

To amplify the signal from our microspheres assay, we used a biotin-labeled anti-streptavidin antibody amplification system. The signal intensity was amplified, after hybridizing the microspheres with their targets (cRNA), by a second staining with a biotin-labeled anti-streptavidin antibody followed by a second streptavidin-R–phycoerythrin staining. Incorporating the antibody amplification step into the assay, as described in “Materials and Methods,” led to an average increase in signal intensity of approximately 5- to 10-fold (Figure 2). The fluorescent signal corresponding to the transcript levels of *Calb3* in control samples increased approximately 4-fold from 68 units to 234 units using 10 μg cRNA, but the fluorescent signal of the sample derived from tissues exposed to EE in the high-dose group (10 μg/kg/day) increased from 980 to 10,632 units by using the amplification step.

**Figure 3:**
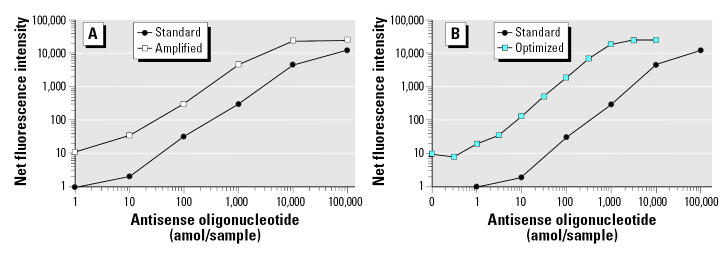
Example of the optimization of the hybridization assay for the quantification of *Calb3.* (*A*) Antisense oligonucleotide for *Calb3,* at various concentrations (1, 10, 100, 1000, 10,000, and 100,000 amol/sample), was hybridized to the specific *Calb3* microspheres coupled to the specific probe for *Calb3,* and the mean fluorescence intensity of at least 100 microspheres was determined (standard). To increase the signal value, equivalent samples of antisense oligonucleotide for *Calb3* were hybridized to the specific *Calb3* microspheres and washed, and then hybridization signal was amplified as indicated in methods (amplified or base). (*B*) The optimization of the hybridization conditions, as indicated in “Materials and Methods,” resulted in a greater increase in sensitivity of the assay (optimized).

To better understand the magnitude of increased sensitivity from the amplification step, we measured the increase in signal obtained with a titration curve of complementary biotinylated oligonucleotide. The saturation concentration is relative to the maximum signal value of the detector in the flow cytometer. In Figure 3A, the titration curve of a biotinylated complementary oligonucleotide for *Calb3* is shown for the comparison of the nonamplified and amplified signal. The assay sensitivity increased approximately 10-fold in the amplified assay compared with the nonamplified assay.

Additional refinements to the assay, including changes in the hybridization buffer, the incubation time, and instrument PMT settings, have led to an additional 10-fold increase in sensitivity. Fluorescent signals are now at least 8- to 20-fold above background, which makes data calculations far more reliable (Figure 3B), for relatively low abundance as well as abundant transcripts.

**Figure 4:**
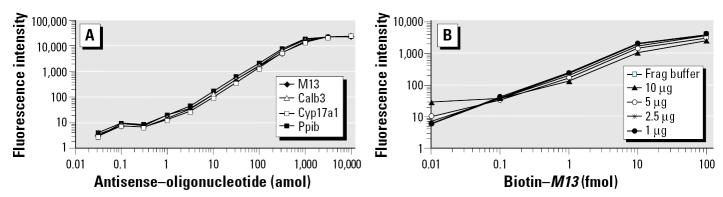
Specificity and dynamic range of detection with amplification. Signal amplification does not change the linearity of the relationship between the amount of target quantified and the magnitude of the signal, even when the analyte is found within a complex mixture of cRNA potential targets. (*A*) Antisense oligonucleotide for *Calb3*, *Cyp17a1*, *Ppib*, and *M13* at various concentrations (1, 10, 100, 1000, 10,000, and 100,000 amol/sample) were mixed and hybridized to the specific *Calb3*-, *Cyp17a1*-, *Ppib*-, and *M13*-specific microspheres in a four-plex format, and the mean fluorescence intensity of at least 100 microspheres was determined after the hybridization signal was amplified as indicated in methods. (*B*) The indicated amounts of *M13* antisense oligonucleotide were mixed with fragmentation buffer or with various amounts of a highly complex cRNA sample, and then hybridized with *M13*-specific microspheres.

The signal amplification steps do not change the linearity of the relationship between the amount of target quantified and the magnitude of the signal regardless of the complexity of the sample or the level of multiplexing (Figure 4A). The signal amplification step allows the simultaneous quantification of multiple analytes in the linear range from 1 up to 1,000 amol. In addition Figure 4B demonstrates that a titration of cRNA from rat uterus/ovaries (1, 2.5, 5, or 10 μg total) has no effect on the sensitivity of the assay for the control biotinylated complementary oligonucleotide for *M13*. As can be seen in Figure 4B, even in the context of the whole population of labeled uterine/ovarian cRNA of the rat, the amplification of the signal does not modify the specificity or the sensitivity of the assay

### Gene expression changes induced by EE in the uterus/ovaries of the prepubertal rat: microarray versus microsphere-based approach.

**Figure 5:**
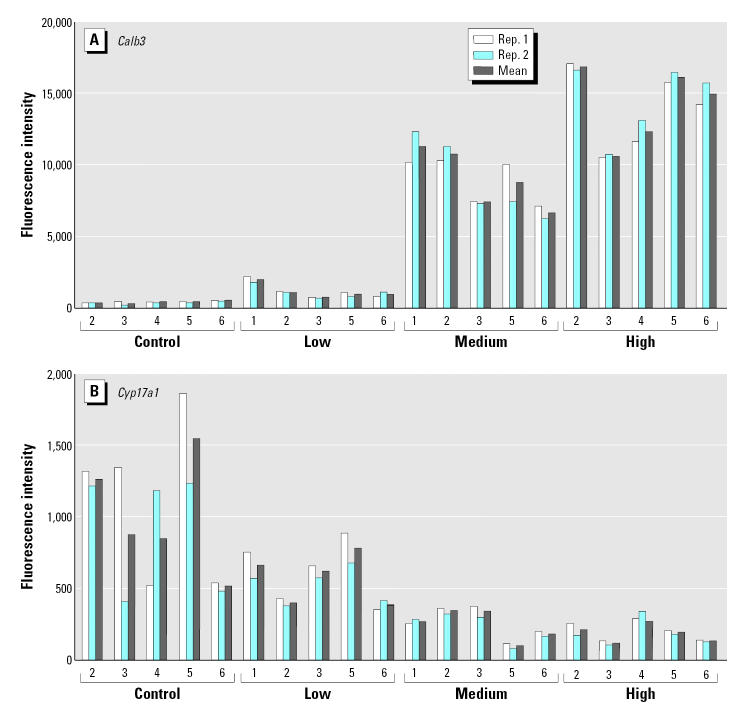
Reproducibility of the assay, exemplified with the determination of the expression levels of (*A*) *Calb3* (up-regulated gene) and (*B*) *Cyp17a1* (down-regulated gene) in five independent samples (biologic replicas), in two independent experiments (Rep. 1 and Rep. 2) with the complete set of samples. Ten micrograms of biotinylated cRNA from control vehicle-treated (control) or EE-exposed tissues (0.1, 1.0, or 10.0 μg/kg/day; low, medium, and high, respectively) was hybridized to microspheres in a five-plex format (with the specific sets for *Calb3*, *Cyp17a1*, *Ppib*, *Hsd11b2*, and *M13*) under the optimized protocol as indicated in “Materials and Methods.” Duplicate results and the means from five samples for each treatment regimen are shown for *Calb3* and *Cyp17a1*. The transcripts are identified in Table 1.

To evaluate the overall performance of the multiplex assay, the expression levels of the 20 selected genes were evaluated under the optimized conditions. After hybridization and amplification of the signal, the expression levels of the 20 genes were measured simultaneously. Replicate measurements from five independent biological samples (biological replicas) from each dose group (0, 0.1, 1, and 10 μg EE/kg/day; control, low, mid, and high dose, respectively) were made. The reproducibility of the assay is represented in Figure 5 with the determination of the expression levels of *Calb3*, an up-regulated gene, and *Cyp17a1*, a down-regulated gene, in independent samples. The comparison of the signal values (arbitrary fluorescent units) obtained from the microarray analysis (Affymetrix) versus the ones obtained from the optimized xMAP assay for four representative transcripts is shown in Table 2. It is clear that there is a high concordance on the hybridization signal values between the two methods, which results in a one-to-one correspondence in the gene expression changes determined by microarray analysis and by the xMAP assay. In both cases, there is a clear dose response for genes that are up-regulated (*C3*, *Scya11*, and *Ehhadh*) or down-regulated (*Star*) by EE exposure. Further, the transcripts that are found at relatively low copy number, up-regulated genes in control samples or down-regulated in high-dose–treated samples, or at a high-copy-number, down-regulated genes in controls or up-regulated genes in EE-treated samples, can be quantified unambiguously.

The results from the evaluation of the 17 target transcripts representing the product of genes whose transcription is regulated by estrogens, as well as two genes not regulated by estrogens (internal controls: vascular α-actin and cyclophilin B), from the uterus/ovaries of the immature rats exposed to 10 μg EE/kg/day (high dose) are shown in Table 3. The Affymetrix microarray data for the indicated genes, and many others, have already been published (Naciff et al. 2003). The relative expression level was determined by calculating the ratio of high-dose EE-treated to control-treated samples. Some experiments (run 1, multiple quantifications in independent samples) were performed at Radix Biosolutions, where the hybridization signal was quantified using a Luminex 100 SP1 software, version 1.7.69. Replicate experiments (run 2) of the same samples were performed at Procter & Gamble, where the hybridization signal was quantified on a Bio-Rad Bio-Plex (Bio-Rad’s version of the Luminex 100) using the Bio-Plex Manager software, version 2.0 . For most of the analytes, the fluorescent signals were highly reproducible, particularly when the analyte represented low or moderately abundant transcripts (Table 3). However, there were some discrepancies with the quantitation of transcripts with high copy number, such as *C3* (complement component 3). The samples read on the Bio-Plex instrument were taken at the high PMT setting to enhance the instrument’s overall sensitivity; however, this setting causes the PMT to reach signal saturation more readily. Thus, for highly expressed genes such as *C3*, signal saturation on the Bio-Plex instrument resulted in discordant data between the replicate experiments. Changing the PMT gain setting of the Bio-Plex (or the Luminex) instrument and rereading the same samples allowed the determination of analytes present in the samples at relatively high copy numbers and did not modify the quantitation of the other analytes (Table 3). A second reading of the same samples shows only a 10% (± 3%) loss of the signal intensity by photobleaching of the reporter (data not shown). Thus, reading the hybridization signal of the same test samples at two PMT settings (low and then high) results in the expansion of the dynamic range of the assay without losing sensitivity.

## Discussion

**Figure 6:**
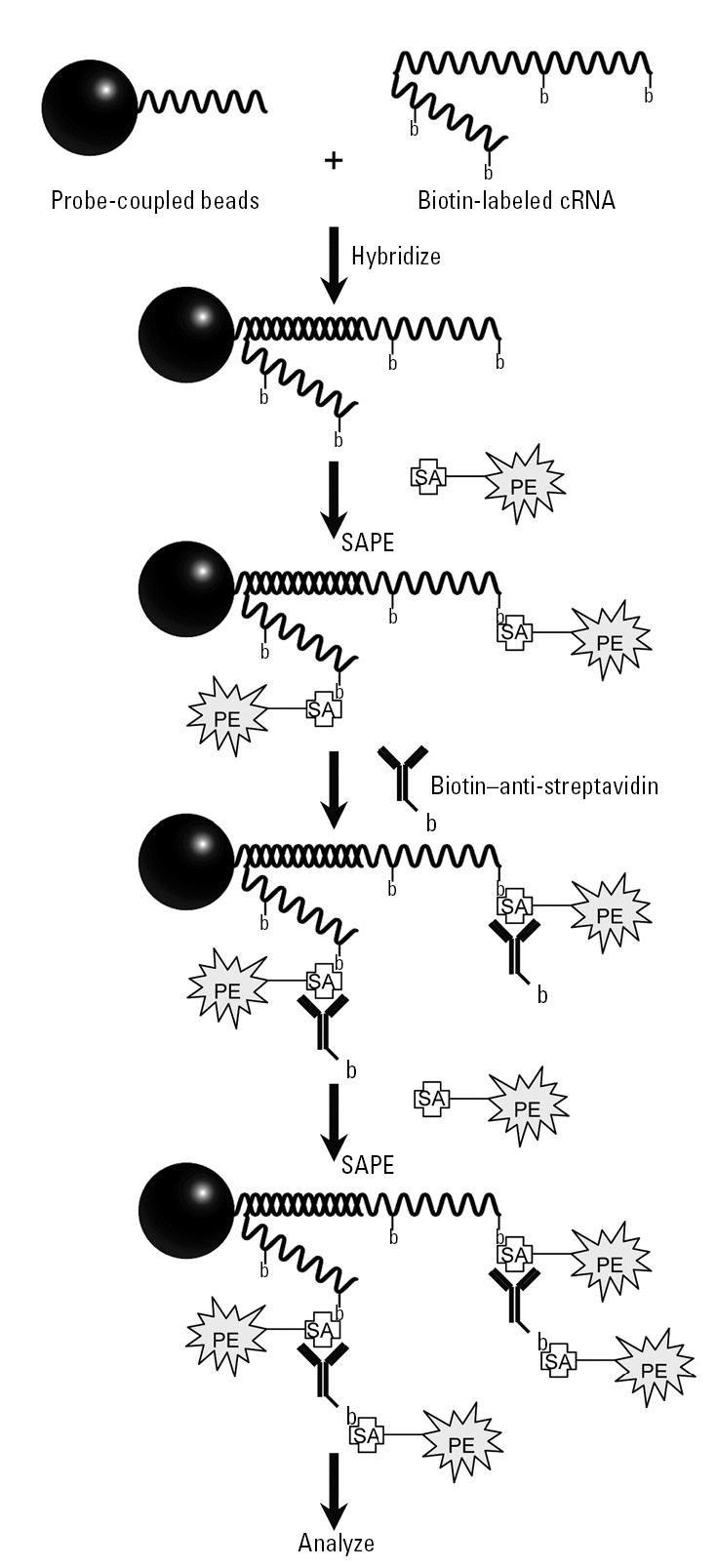
Schematic representation of the microsphere-based high-throughput gene expression assay. Fluorescent microspheres with covalently bound oligonucleotides specific to the cRNA to be quantified are incubated with cRNA and biotin (b) labeled. Hybridized cRNA is revealed by SAPE [phycoerythrin (PE)-conjugated streptavidin (SA)]. The signal is amplified by a second stain using the biotinylated anti-streptavidin, followed by a third staining step with SAPE.

We have developed a high-throughput gene expression profiling assay using fluorescent labeled microspheres coupled with gene-specific oligonucleotides to evaluate the effects of estrogen exposure in the prepubertal rat. Substantial improvements on existing bead-based assays have been made that result in an assay that is highly correlated in relative gene expression changes determined by established microarray technologies. These improvements include an anti-streptavidin amplification method that produces a 10-fold increase as well as changes to the hybridization buffer, assay kinetics, and hybridization temperature. Using these improvements, a detection limit of 1 amol of complementary RNA has been achieved, allowing the analysis of rare messages in complex cRNA samples using as little as 2.5 μg starting material. This assay offers increased throughput with decreased costs compared with existing microarray technologies, allowing the determination of gene expression changes specifically, rapidly, and with high sensitivity and resolution. This system is a rapid multiplexed assay platform that quantifies up to 100 distinct analytes simultaneously in a single sample in a 96-well plate format. The microsphere-based assay format provides the flexibility to rapidly customize the specific genes being examined in an assay simply by adding or removing individual bead sets from the assay’s mix. Our data demonstrate that this approach can be used to create a high-throughput screening assay to identify potential gene expression changes induced by a given treatment, such as the one used in this study to identify chemicals with estrogenic activity. The multiplexing capability offers the opportunity to screen large numbers of chemicals to determine their potential therapeutic and/or toxic properties, in a cost- and animal-effective manner using a customized set of genes. The complete optimized protocol is described in Table 4. The schematic representation of the microsphere-based high-throughput gene expression assay is shown in Figure 6.

The method presented in this article could be used in a variety of applications related to assaying for hybridization of a target polynucleotide to an oligonucleotide probe and amplification of a signal generated thereby. It can be used as a first- or second-tier assay to extract data of biomedical relevance for a wide range of applications. For example, it can be adapted to evaluate specific gene expression changes used as “biomarkers” to identify desired (therapeutic) versus undesired (toxic) outcomes in the identification of different pathogens present as potential contaminants of food stuffs, drinking water or even in the air, or in the determination of a pathological state (e.g., see Bau et al. 2002; Rhodes et al. 2004; Zhang et al. 2002).

Methods for the detection of specific transcripts have advanced at a relative fast pace. However, they have some drawbacks, including high background noise, extended time and labor requirements, lack of specificity, lack of sensitivity, and a high entry and operation cost. The method we have developed offers a cost-efficient system that demonstrates low background noise, high specificity and sensitivity, and after cRNA labeling can be completed in as little as 4 hr. In the available literature, there are some approaches to identify specific gene expression changes; however, they have some limitations overcome by the assay here described. Particularly, Yang et al. (2001) reported the use of microspheres linked to a capture probe that has sequence complementary to a first segment of a sequence of a single-strand target nucleic acid. However, this method is not as sensitive and can detect only relatively abundant transcripts. On a different approach, Fuja et al. (2004) described a four-plex assay to determine the expression of four genes using carboxyl–polystyrene particles of four sizes. The primary limitation with this approach is the limited number of different-sized microspheres to expand the assay to greater than four transcripts in a single reaction. With a different approach Georgieva et al. (2002) described a method based on magnetic-activated cell separation followed by a nested reverse-transcriptase–PCR to quantify tyrosinase and MART-1 mRNA. The data from these authors indicated a detection rate comparable to that of other established total RNA extraction methods. However, not all the results were concordant, and the assay does not seem to be very reliable. On a theoretical basis Hug and Schuler (2003) proposed a method to calculate the number of molecules of a single mRNA species in a complex mRNA preparation by cloning tagged nucleic acid molecules onto the surface of microbeads and amplifying them, followed by their quantification. However, to our knowledge this method has not been implemented. For another application of microsphere-based assay, Spiro and Lowe (2002) developed a procedure for multiplexed quantification of PCR products using the bead method in a manner that can lead to high-throughput testing, to determine microorganisms from contaminated groundwater. The major limitations of their approach are the amount of starting material they could quantify and the requirement for PCR amplification.

The assay we have developed overcomes the various limitations found with other methods and is highly flexible compared with current methods. In addition the duration of the assay hybridization step can vary from a minimum of 1 hr up to 18 hr. Because hybridization is between single-strand cRNA and single-strand oligonucleotide capture probes, increasing the hybridization time up to 18 hr can double the sensitivity of the assay. The enhancement of sensitivity described here permits the amount of input material (cRNA) to be as little as 1 μg in a complex mixture of cRNAs or polynucleotides such as total RNA, or mRNAs, according to the desired application. The oligonucleotide(s) used to create the assay may be preoptimized, as we have done, or the multiplexing capability of the system allows the optimized oligonucleotide to be selected empirically. For example, one or more oligonucleotides specific for the same gene may be coupled to different microsphere sets, combined, and subjected to a hybridization-based assay to determine the best signal (specificity and sensitivity) for the desired set of analytes.

Although in this study the amplified signal is detected using a flow cytometer, other means to detect the amplified signal are suitable and within the scope of the present method. The Luminex-based analyzers use standard 96-well plate formats, thereby providing this assay platform with higher sample throughput capabilities than other standard array formats. In addition, the use of the standard microtiter plate format makes the system amenable to automation.

In summary, we have developed an assay to quantify specific gene expression changes in a complex background with high sensitivity and specificity. We have made several improvements on existing bead-based assays that provide results that highly correlate with standard microarray technologies. We have achieved detection levels down to 1 amol (10^−18^ mol), detecting rare messages in complex cRNA samples, using as little as 2.5 μg. This assay offers sensitivity greater than that achieved by Affymetrix GeneChip microarrays with identical sample preparations. The assay can analyze up to 100 analytes simultaneously (validated with 20), is highly flexible (add/subtract any given analyte by adding or removing specific microsphere sets), and offers significant time savings over QRT–PCR and it has high throughput capabilities on a standard 96-well format (scaleable to 384-well format).

## Figures and Tables

**Table 1:** Genes selected for the bead-based high-throughput gene expression assay (20-plex format).

Accession no.	Gene name	Gene symbol	Probe sequence
AB006007	steroidogenic acute regulatory protein	*Star*	5′-ACGTGGCTGCTCAGTATTGACCTCA-3′
AF022147	uterus-ovary specific putative transmembrane protein or CUB and zona pellucida-like domains 1	*Cuzd1*	5′-CGTCATGCTCGTATCACAGCCTCAG-3′
K03249	peroxisomal enoyl-CoA-hydrotase-3-hydroxyacyl-CoA	*Ehhadh*	5′-TGGATCTGTAACACATTGAGTTCAA-3′
L00191	fibronectin 1	*Fn1*	5′-TGGCCACACCTACAACCAGTATACA-3′
L11007	cyclin-dependent kinase 4	*Cdk4*	5′-TGGAGTGTTGGCTGTATCTTCGCAG-3′
L26292	FSH-regulated protein or Kruppel-like factor 4	*Klf4*	5′-TTTGTCTTCCGATCTACATTTATGA-3′
M14656	osteopontin or secreted phosphoprotein 1	*Spp1*	5′-AGAGAGTTCATCTTCTGAGGTCAAT-3′
M29866	complement component C3	*C3*	5′-GTCAAGGTCTACTCCTACTACAATC-3′
M31837	insulin-like growth factor-binding protein 3	*Igfbp3*	5′-TACAGAGCTTTCCTTGAGAGCACAA-3′
M57664	cretine kinase-B	*Ckb*	5′-GTTTTTGATGTCTCCAACGCTGACC-3′
M86389	heat shock protein 27	*Hspb1*	5′-ATGAGTGGTCTCAGTGGTTCAGCTC-3′
X82396	cathepsin B	*Ctsb*	5′-GCTGCACCTTGAAGCTAGTCACTTC-3′
Y08358	eotaxin or small inducible cytokine subfamily A11	*Scya11*	5′-GAAATAGGGTCTCACTGTATCACCC-3′
X06801	vascular alpha-actin	*VaACTIN*	5′-TACTGCTGAGCGTGAGATCGTCCGT-3′
S64044	progesterone receptor	*Pgr*	5′-TTTGCACCTGATCTAATCCTGAATG-3′
V00604	bacteriophage M13	*M13*	5′-AAGCAACCATAGTACGCGCCCTGTA-3′
K00994	intestinal calcium-binding protein or calbindin 3	*Calb3*	5′-CGACACCACCTACTGATTGAATCCT-3′
M21208	cytochrome P450, family 17, subfamily a, polypeptide 1	*Cyp17a1*	5′-CTCAACACCCACAGTACAATCTTAG-3′
U22424	11-beta-hydroxylsteroid dehydrogenase type 2	*Hsd11b2*	5′-TCATGAGACCATGTATACCCTACCA-3′
AF071225	cyclophilin B or peptidylprolyl isomerase B	*Ppib*	5′-AGCAAGTTCCATCGTGTCATCAAGG-3′

Gene annotations are from GenBank (http://www.ncbi.nih.gov/GenBank) with the exception of *VaACTIN* and *M13*.

**Table 4:** Optimized protocol.

For duplicate wells
2.5 μg cRNA target per well
10 μL stock cRNA (0.5 μg/μL)
40 μL 1/2 × TMAC hybridization buffer containing 100 amol *M13* oligo hybridization control spike
Microsphere mixture: 800 μL (enough for 20 samples)
2 μL each bead stock used in 20-plex (stock = 10^7^ microspheres/mL)
760 μL 1/2 × TMAC hybridization buffer
Procedure
1. Add 25 μL diluted cRNA target.
2. Add 25 μL bead mix to each well according to step 1 above.
3. Incubate at 95°C for 2 min.
4. Transfer plate to thermo mixer, cover, and hybridize 3 hr up to overnight at 48°C while shaking at 500 rpm.
5. Spin samples in centrifuge at 2,250 × *g* for 2 min; flick and tap off solution.
6. Wash microspheres with 100 μL 1/2 × TMAC.
7. Spin samples in centrifuge at 2250 × *g* for 2 min; flick and tap off solution.
8. Wash microspheres with 100 μL PBS–BSA.
9. Spin samples in centrifuge at 2,250 × *g* for 2 min; flick and tap off solution.
10. Add 50 μL SAPE stain mix; shake at 500 rpm at 25°C for 15 min.
11. Spin samples in centrifuge at 2,250 × *g* for 2 min; flick and tap off solution.
12. Wash microspheres with 100 μL PBS–BSA.
13. Spin samples in centrifuge at 2250 × *g* for 2 min; flick and tap off solution.
14. Add 50 μL anti-streptavidin and goat IgG; shake at 500 rpm at 25°C for 60 min.
15. Spin samples in centrifuge at 2,250 × *g* for 2 min; flick and tap off solution.
16. Wash microspheres with 100 μL PBS–BSA.
17. Spin samples in centrifuge at 2,250 × *g* for 2 min; flick and tap off solution.
18. Add 50 μL SAPE; shake at 500 rpm at 25°C for 15 min.
19. Spin samples in centrifuge at 2,250 × *g* for 2 min; flick and tap off solution.
20. Wash microspheres with 100 μL PBS–BSA.
21. Spin samples in centrifuge at 2,250 × *g* for 2 min; flick and tap off solution.
22. Resuspend in 65 μL PBS–BSA and read on low- and then high-PMT settings of the instrument.

**Table 2:** Signal values (arbitrary fluorescent units) obtained from the microarray (Affymetrix) versus the xMAP.

	Microarray				xMAP			
Gene transcript	Control	0.5 μg EE	1 μg EE	10 μg EE	Control	0.5 μg EE	1 μg EE	10 μg EE
*Star*	2,558	2,626	370 (A)	296 (A)	1,169	1,366	384	397
*Ehhadh*	210 (A)	325 (A)	1,294	1,267	181	210	1,147	1,027
*Scya11*	293 (A)	855 (M)	4,542	10,419	843	1,296	5,039	10,210
*C3*	74 (A)	310 (A)	39,138	32,320	779	1,169	27,547	26,761

The average signal value of the indicated transcripts obtained from the uterus/ovaries from five females exposed to vehicle control or the indicated doses of EE for 4 days (μg EE/kg/day) as indicated in Naciff et al. (2003). Transcripts for which an absent (A) or marginal (M) call was determined (MAS 5.0, Affymetrix) in all the samples (*n* = 5) are noted even though there is a signal value.

**Table 3:** Relative expression level^a^ of selected genes determined by microarray compared with xMAP technology.

	Gene expression average fold change (*n* = 5)			
Gene symbol	Affymetrix microarray^b^	xMAP run 1 (high PMT)	xMAP run 2 (high PMT)	xMAP run 2 (low PMT)
VaACTIN	1.0	1.0	1.0	1.0
Ppib	1.0	1.1	1.1	1.1
Hsd11b2	4.0	3.3	4.2	3.9
Ctsb	2.6	3.8	3.2	3.6
Ckb	2.6	3.5	2.8	3.4
C3	295.0	144.0	36.2	100.2
Ehhadh	6.0	3.9	6.5	5.5
Scya11	35.5	14.4	12.7	14.6
Hspb1	2.1	2.2	2.0	2.1
Pgr	2.1	1.8	1.8	1.9
Calb3	10.5	46.5	10.7	35.2
Fn1	2.6	3.4	2.7	3.2
Cuzd1	12.9	38.3	16.7	38.2
Klf4	3.3	2.4	2.4	2.4
Star	−8.7	−3.2	−3.1	−2.6
Igfbp3	−4.4	−2.5	−2.8	−2.6
Cyp17a1	−15.1	−6.1	−4.9	−4.8
Spp1	−3.5	−2.3	−2.3	−2.0
Cdk4	−1.6	−1.2	−1.2	−1.2

a The relative expression level is represented by the ratio of high-dose EE-treated to control-treated samples. Run 1 was done at Radix Biosolutions (read on a Luminex 100 flow cytometer using SP1 software, version 1.7), and run 2 was done at Procter & Gamble (read on the Bio-Rad Bio-Plex using the Bio-Plex Manager software, version 2.0).

b The Affymetrix microarray data for the indicated genes, and many others, have been published (Naciff et al. 2003).
